# Supplementary material for: Medial temporal atrophy in preclinical dementia: Visual and automated assessment during six year follow-up
Source: Neuroimage Clin. 2020 Jun 10;27:102310. doi: 10.1016/j.nicl.2020.102310 (PMC7317671; doi:10.1016/j.nicl.2020.102310)
Supplement: Supplementary data [file mmc1.pdf]

Supplementary Data - Medial temporal atrophy in preclinical dementia: visual and automated assessment during six year follow-up

Gustav Mårtensson[1,*], Claes Håkansson[2], Joana B. Pereira[1], Sebastian Palmqvist[3,4], Oskar Hansson[3,4], Danielle van Westen[2,5,†], and Eric Westman[1,6,†]

[*]Corresponding author: gustav.martensson@ki.se
[†]Shared last author.
[1]Department of Neurobiology, Care Sciences and Society, Karolinska Institute, Stockholm, Sweden.
[2]Diagnostic Radiology, Institution for Clinical Sciences, Lund University, Lund, Sweden.
[3]Clinical Memory Research Unit, Department of Clinical Sciences in Malmö, Lund University, Lund, Sweden.
[4]Memory Clinic, Skåne University Hospital, Malmö, Sweden.
[5]Image and Function, Skåne University Hospital, Lund, Sweden.
[6]Department of Neuroimaging, Centre for Neuroimaging Sciences, Institute of Psychiatry, Psychology and Neuroscience, King's College London, London, UK.

A Supplementary data

As additional information we provide:

- Box- and scatterplots of the MTA ratings vs. neuropsychological tests (Fig S1).
- Confusion matrices for all rating sets (Tables S1-S3).
- Atrophy rates' association with phosphorylated tau levels in the CSF (Fig. S2).
- Corresponding plots to Figure 3 in main study (medial temporal lobe measures as a function of age) for the right hemisphere (Fig. S3), normalized with total intracranial volume (Fig. S4), and averaged bilaterally (Fig. S7).
- Visual examples of the MTA rating slice for four subjects (Fig. S5).
- Mean and standard deviations of the MTA scores per timepoint, stratified according to CSF biomarker profile and cognitive status (Fig. S6).
- Corresponding tables to Table 3 in main study (baseline and annual changes for all medial temporal lobe measures) for subcortical volumes normalized with total intracranial volume (Table S4), and averaged bilaterally (Table S5).

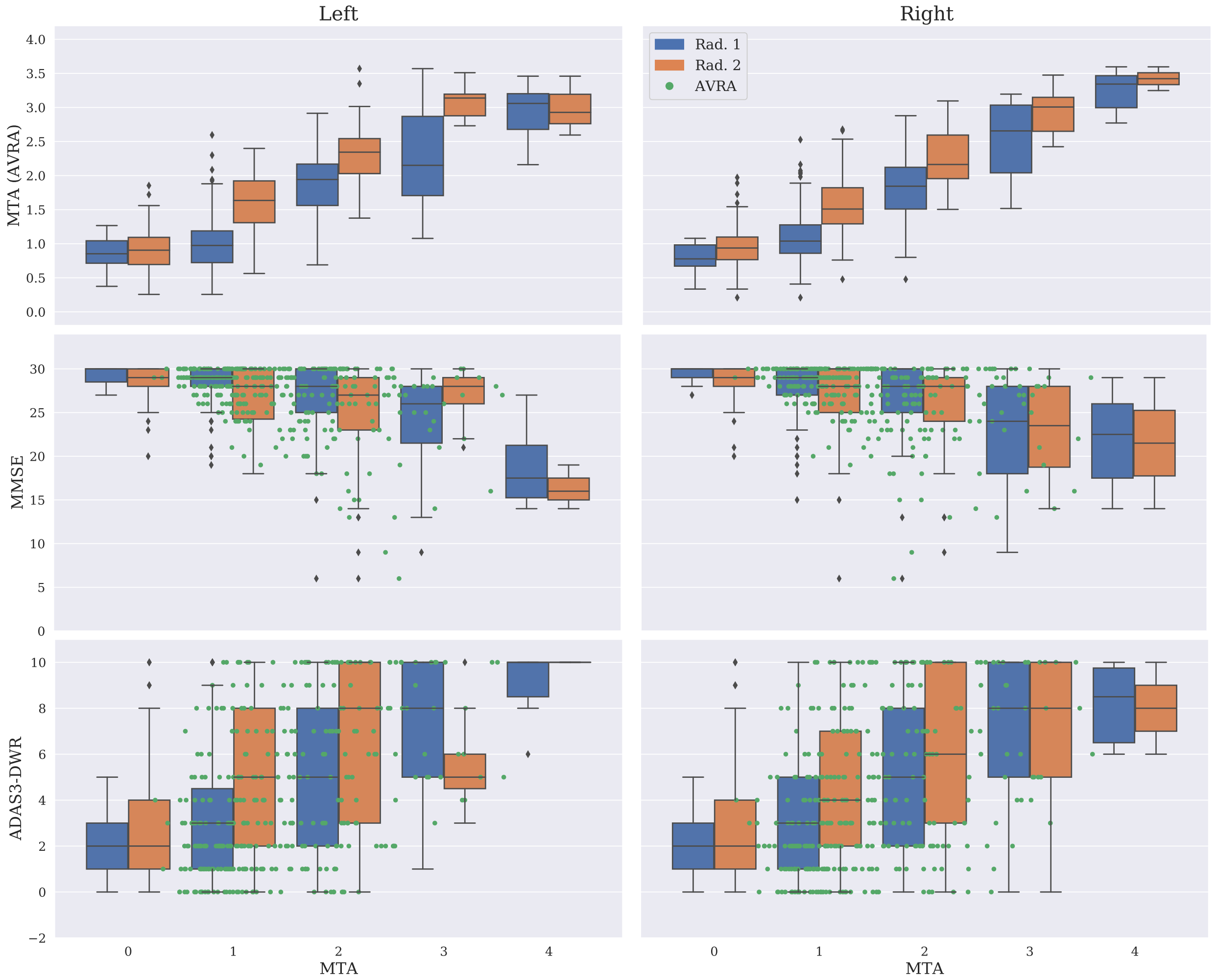

Figure S1: Boxplots of the radiologists' MTA ratings and corresponding AVRA ratings (*top*), MMSE score (*middle*), and ADAS3-DWR score (*bottom*). The green dots show AVRA's MTA ratings for neuropsychological tests.

Table S1: Confusion matrices for left and right MTA ratings between Rad. 1 and Rad. 2.

Left

		Rad. 1				
		0	1	2	3	4
Rad. 2	0	19	145	7	4	0
	1	0	36	73	9	0
	2	0	2	41	19	1
	3	0	0	2	7	2
	4	0	0	0	0	3

Right

		Rad. 1				
		0	1	2	3	4
Rad. 2	0	19	128	10	0	0
	1	0	56	71	3	0
	2	0	4	48	12	1
	3	0	0	1	12	3
	4	0	0	0	0	2

Table S2: Confusion matrices for left and right MTA ratings between Rad. 1 and AVRA.

Left

		Rad. 1				
		0	1	2	3	4
AVRA	0	1	5	0	0	0
	1	18	158	26	5	0
	2	0	19	83	19	1
	3	0	1	14	13	5
	4	0	0	0	2	0

Right

		Rad. 1				
		0	1	2	3	4
AVRA	0	1	6	1	0	0
	1	18	157	28	0	0
	2	0	24	85	10	0
	3	0	1	16	17	5
	4	0	0	0	0	1

Table S3: Confusion matrices for left and right MTA ratings between Rad. 2 and AVRA.

Left

		Rad. 2				
		0	1	2	3	4
AVRA	0	6	0	0	0	0
	1	162	44	1	0	0
	2	7	74	41	0	0
	3	0	0	20	10	3
	4	0	0	1	1	0

Right

		Rad. 2				
		0	1	2	3	4
AVRA	0	7	1	0	0	0
	1	143	60	0	0	0
	2	7	66	45	1	0
	3	0	3	20	15	1
	4	0	0	0	0	1

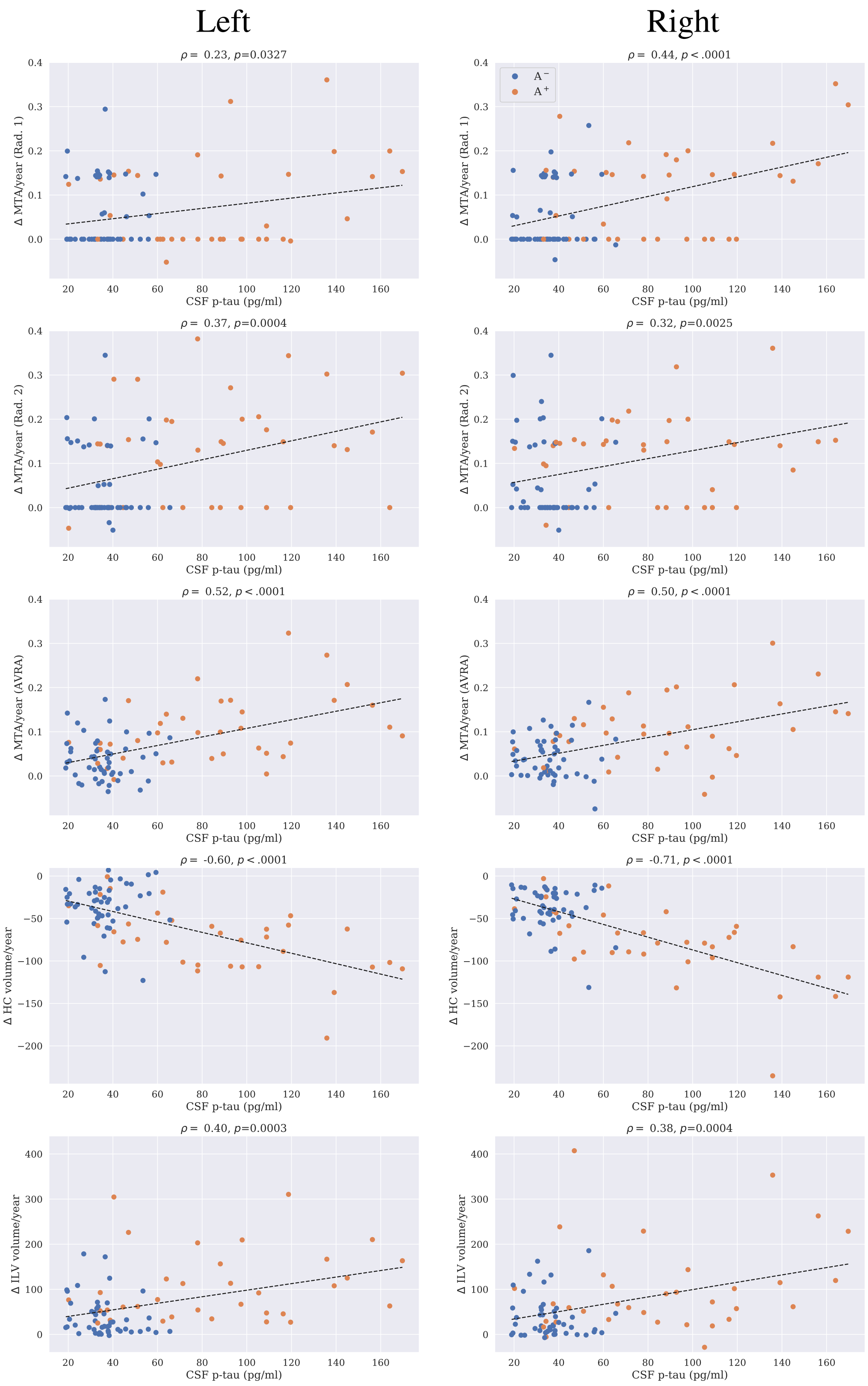

Figure S2: Scatterplot of relationship between CSF p-tau level at baseline and annual change in (*from the top*) MTA ratings by Rad. 1; MTA ratings by Rad. 2; MTA ratings by AVRA; Hippocampal (HC) volumes; Inferior lateral ventricles (ILV) volumes. The blue dots show cases that are amyloid negative (A^-) and orange amyloid positive (A^+). The title of the plots show the Pearson correlation (ρ) and corresponding p-value. The dashed black lines show the linear regression model fit.

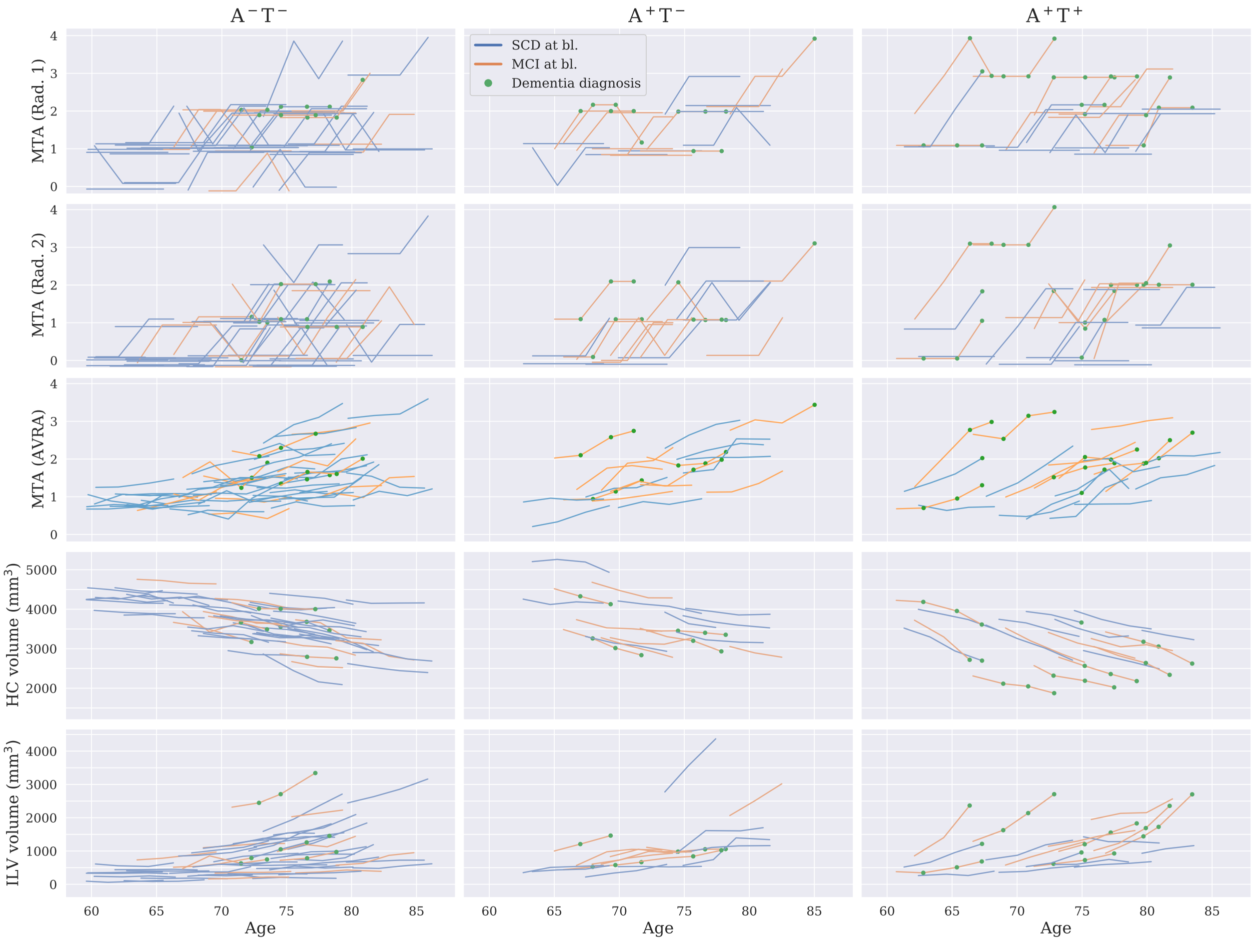

Figure S3: Medial temporal lobe measures of the **right hemisphere** plotted against age at scan time for different combinations of Aβ (A) and phosphorylated tau (T) abnormality. *From the top*: MTA ratings by Rad. 1; MTA ratings by Rad. 2; MTA ratings by AVRA; Hippocampal (HC) volumes; Inferior lateral ventricles (ILV) volumes. Orange and blue lines show individual trajectories for SCD and MCI patients, respectively. The green dots show if a patient was diagnosed with dementia at the given timepoint. A small random offset ($|\varepsilon| < .175$) has been added to each individual's Rad. 1 and Rad. 2 ratings to make it easier to follow overlapping trajectories.

Table S4: Average baseline (bl) MTA ratings and volumes **normalized with total intracranial volume** (TIV), and average annual change for individuals with different CSF statuses. Rows in bold denote entries where the whole CSF group was considered (i.e. SCDs and MCIs), and 'SCD/MCI only' refers to the subset of SCD/MCI subjects within the CSF group. ΔMTA/year refers to the average annual change in MTA score of the study participants. The reported p-values were computed using Kruskal-Wallis H-test to test the null-hypothesis that the population medians of all CSF groups were equal. Applying a Bonferroni correction to a significance level of $\alpha = 0.05$ means rejecting the null-hypothesis for $p < \frac{\alpha}{m} = \frac{.05}{66} \approx .00076$, where m is the number of statistical comparisons.

	A^-T^-		A^+T^-		A^+T^+		p-value	
Measure	Left	Right	Left	Right	Left	Right	Left	Right
**Rad. 1: MTA at bl.**	**1.17 ± 0.66**	**1.17 ± 0.63**	**1.56 ± 0.68**	**1.28 ± 0.45**	**1.67 ± 0.78**	**1.43 ± 0.58**	**0.0282**	**0.2783**
SCD only	1.07 ± 0.63	1.10 ± 0.61	1.38 ± 0.48	1.38 ± 0.48	1.27 ± 0.45	1.18 ± 0.39	0.2492	0.3542
MCI only	1.50 ± 0.65	1.42 ± 0.64	1.70 ± 0.78	1.20 ± 0.40	2.10 ± 0.83	1.70 ± 0.64	0.2835	0.1948
**Rad. 1: ΔMTA/year**	**0.05 ± 0.09**	**0.05 ± 0.08**	**0.04 ± 0.07**	**0.07 ± 0.09**	**0.09 ± 0.11**	**0.12 ± 0.10**	**0.0255**	**<.0001**
SCD only	0.05 ± 0.09	0.04 ± 0.08	0.04 ± 0.06	0.03 ± 0.05	0.07 ± 0.10	0.08 ± 0.11	0.8090	0.3825
MCI only	0.05 ± 0.09	0.06 ± 0.08	0.04 ± 0.07	0.11 ± 0.10	0.11 ± 0.11	0.16 ± 0.07	0.0016	<.0001
**Rad. 2: MTA at bl.**	**0.50 ± 0.71**	**0.56 ± 0.79**	**0.61 ± 0.76**	**0.50 ± 0.69**	**0.62 ± 0.79**	**0.81 ± 0.85**	**0.7581**	**0.3620**
SCD only	0.36 ± 0.65	0.52 ± 0.79	0.50 ± 0.71	0.62 ± 0.70	0.27 ± 0.45	0.45 ± 0.66	0.8081	0.8203
MCI only	1.00 ± 0.71	0.67 ± 0.75	0.70 ± 0.78	0.40 ± 0.66	1.00 ± 0.89	1.20 ± 0.87	0.6051	0.0902
**Rad. 2: ΔMTA/year**	**0.05 ± 0.08**	**0.06 ± 0.09**	**0.11 ± 0.10**	**0.13 ± 0.07**	**0.15 ± 0.12**	**0.13 ± 0.12**	**<.0001**	**<.0001**
SCD only	0.06 ± 0.09	0.06 ± 0.09	0.04 ± 0.07	0.10 ± 0.06	0.11 ± 0.12	0.10 ± 0.10	0.0155	0.0039
MCI only	0.03 ± 0.07	0.07 ± 0.09	0.16 ± 0.10	0.14 ± 0.07	0.19 ± 0.10	0.17 ± 0.13	<.0001	0.0033
**AVRA: MTA at bl.**	**1.26 ± 0.58**	**1.26 ± 0.56**	**1.39 ± 0.71**	**1.40 ± 0.64**	**1.20 ± 0.58**	**1.28 ± 0.64**	**0.6503**	**0.5989**
SCD only	1.18 ± 0.55	1.24 ± 0.55	1.10 ± 0.50	1.33 ± 0.69	1.02 ± 0.43	1.01 ± 0.50	0.7771	0.3942
MCI only	1.54 ± 0.60	1.34 ± 0.60	1.62 ± 0.77	1.46 ± 0.58	1.39 ± 0.65	1.57 ± 0.65	0.8216	0.6186
**AVRA: ΔMTA/year**	**0.04 ± 0.04**	**0.04 ± 0.04**	**0.07 ± 0.05**	**0.08 ± 0.05**	**0.13 ± 0.08**	**0.11 ± 0.08**	**<.0001**	**<.0001**
SCD only	0.04 ± 0.05	0.04 ± 0.04	0.07 ± 0.05	0.07 ± 0.05	0.11 ± 0.09	0.09 ± 0.08	<.0001	<.0001
MCI only	0.04 ± 0.04	0.06 ± 0.04	0.07 ± 0.05	0.09 ± 0.05	0.15 ± 0.07	0.14 ± 0.07	<.0001	<.0001
**HC vol/TIV at bl. (10^{-5})**	**231 ± 31**	**238 ± 33**	**224 ± 33**	**232 ± 31**	**209 ± 32**	**216 ± 33**	**0.1192**	**0.0971**
SCD only	236 ± 30	241 ± 32	245 ± 30	247 ± 30	226 ± 22	235 ± 23	0.5128	0.6829
MCI only	213 ± 26	230 ± 32	206 ± 24	220 ± 26	194 ± 31	199 ± 31	0.5562	0.1851
**Δ(HC/TIV)/year (10^{-5})**	**-2.3 ± 1.7**	**-2.5 ± 1.7**	**-3.3 ± 1.9**	**-3.4 ± 1.9**	**-6.0 ± 2.4**	**-6.3 ± 2.8**	**<.0001**	**<.0001**
SCD only	-2.2 ± 1.7	-2.3 ± 1.6	-2.4 ± 1.6	-2.8 ± 1.3	-5.2 ± 1.6	-5.7 ± 1.8	<.0001	<.0001
MCI only	-2.6 ± 1.6	-3.3 ± 1.6	-4.1 ± 1.8	-3.8 ± 2.1	-6.7 ± 2.7	-6.9 ± 3.4	<.0001	<.0001
**ΔHC/year (%/year)**	**-1.0 ± 0.9**	**-1.1 ± 0.8**	**-1.6 ± 1.0**	**-1.5 ± 0.9**	**-2.9 ± 1.0**	**-2.9 ± 1.2**	—	—
**ILV vol/TIV at bl. (10^{-5})**	**47 ± 30**	**44 ± 29**	**61 ± 39**	**50 ± 30**	**52 ± 25**	**51 ± 24**	**0.2415**	**0.4393**
SCD only	43 ± 28	42 ± 27	46 ± 25	47 ± 36	45 ± 16	42 ± 23	0.6363	0.9979
MCI only	61 ± 31	51 ± 35	75 ± 44	51 ± 24	59 ± 29	59 ± 23	0.7281	0.4784
**Δ(ILV/TIV)/year (10^{-5})**	**2.3 ± 2.5**	**2.4 ± 2.7**	**4.9 ± 3.9**	**4.9 ± 5.0**	**7.5 ± 5.0**	**6.9 ± 6.4**	**<.0001**	**<.0001**
SCD only	2.3 ± 2.7	2.2 ± 2.8	4.1 ± 3.1	5.6 ± 6.0	5.6 ± 5.3	3.1 ± 2.9	<.0001	0.0001
MCI only	2.4 ± 1.7	2.8 ± 2.1	5.6 ± 4.5	4.2 ± 3.7	9.1 ± 3.9	10.4 ± 6.8	<.0001	<.0001
**ΔILV/year (%/year)**	**4.8 ± 4.0**	**4.5 ± 3.9**	**7.9 ± 4.3**	**9.9 ± 7.0**	**14.7 ± 9.0**	**13.9 ± 10.0**	—	—
**ΔMMSE/year**	**-0.15 ± 0.47**		**-0.49 ± 0.70**		**-1.13 ± 1.02**		**<.0001**	
SCD only	-0.05 ± 0.30		-0.19 ± 0.34		-0.87 ± 1.05		<.0001	
MCI only	-0.53 ± 0.71		-0.74 ± 0.82		-1.41 ± 0.92		<.0001	
**ΔADAS-DWR/year**	**-0.04 ± 0.38**		**0.14 ± 0.40**		**0.39 ± 0.50**		**<.0001**	
SCD only	-0.03 ± 0.31		0.01 ± 0.40		0.49 ± 0.62		<.0001	
MCI only	-0.07 ± 0.57		0.25 ± 0.36		0.28 ± 0.26		0.0003	

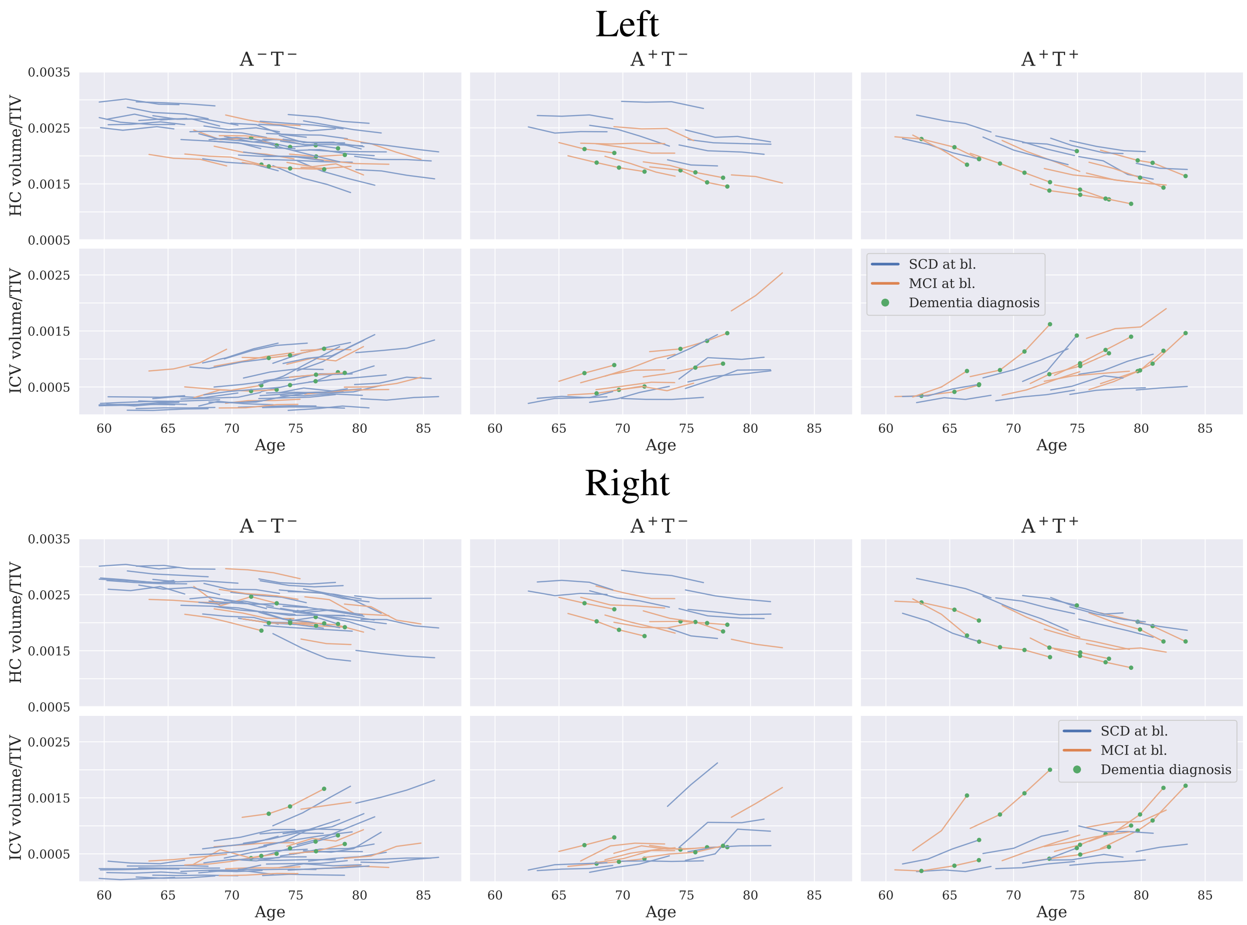

Figure S4: Hippocampal (HC) and inferior lateral ventricle (ILV) volumes normalized with total intracranial volume (TIV) plotted against age at scan time for different combinations of Aβ (A) and phosphorylated tau (T) abnormality. The two top rows show the measures of the left hemisphere, and the two bottom rows the right hemisphere. Orange and blue lines show individual trajectories for SCD and MCI patients, respectively. The green dots show if a patient was diagnosed with dementia at the given timepoint.

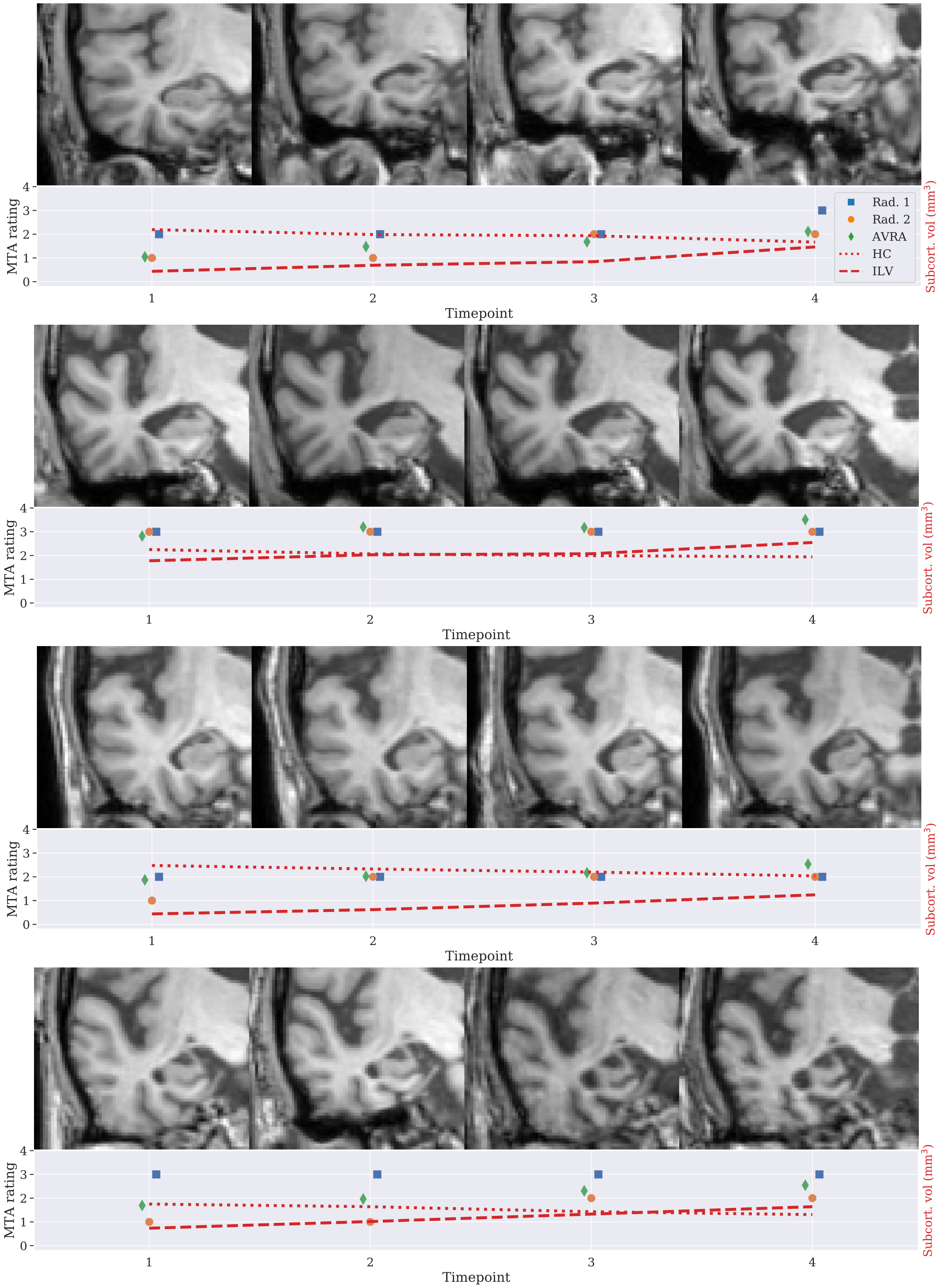

Figure S5: Rating slices at each timepoint of four study participants and corresponding MTA ratings and MTL volumes.)

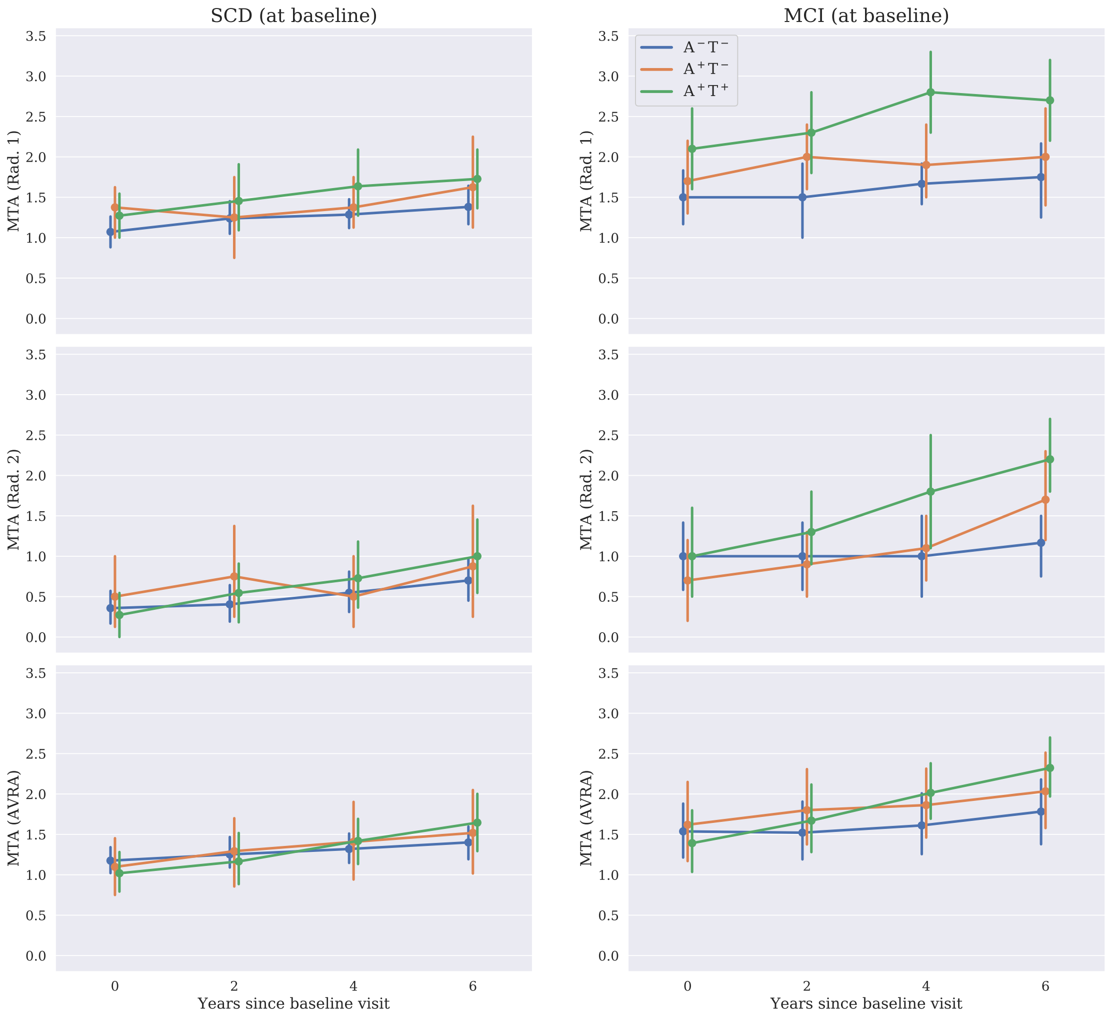

Figure S6: Mean and standard deviations of the left MTA ratings given at each timepoint by Rad. 1 (*top*), Rad. 2 (*middle*), and AVRA (*bottom*) for the different CSF biomarker profiles. The first column shows the progression of subjects who were SCD at baseline, and the second column the subjects with MCI.

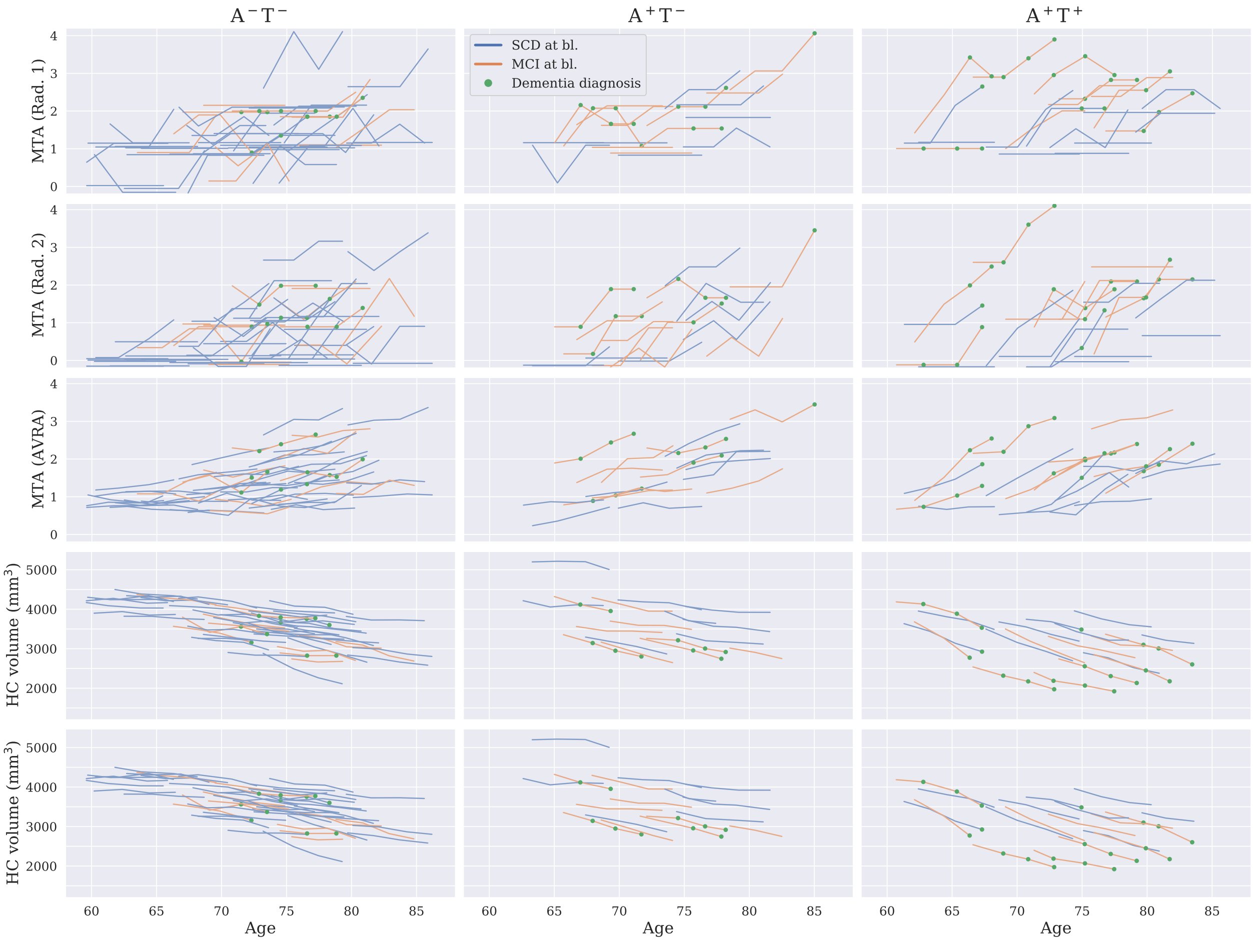

Figure S7: Medial temporal lobe measures **averaged bilaterally** of plotted against age at scan time for different combinations of Aβ (A) and phosphorylated tau (T) abnormality. *From the top*: MTA ratings by Rad. 1; MTA ratings by Rad. 2; MTA ratings by AVRA; Hippocampal (HC) volumes; Inferior lateral ventricles (ILV) volumes. Orange and blue lines show individual trajectories for SCD and MCI patients, respectively. The green dots show if a patient was diagnosed with dementia at the given timepoint. A small random offset ($|\varepsilon| < .175$) has been added to each individual's Rad. 1 and Rad. 2 ratings to make it easier to follow overlapping trajectories.

Table S5: Mean baseline (bl) MTA ratings and volumes, and mean annual change for individuals with different CSF statuses of the different measures *averaged bilaterally*. Rows in bold denote entries where the whole CSF group was considered (i.e. SCDs and MCIs), and 'SCD/MCI only' refers to the subset of SCD/MCI subjects within the CSF group. ΔMTA/year refers to the average annual change in MTA score of the study participants. The reported p-values were computed using Kruskal-Wallis H-test to test the null-hypothesis that the population medians of all groups were equal. Applying a Bonferroni correction to a significance level of $\alpha = 0.05$ means rejecting the null-hypothesis for $p < \frac{\alpha}{m} = \frac{.05}{36} \approx .0014$, where m is the number of statistical comparisons.

	A^-T^-	A^+T^-	A^+T^+	p-value
**Rad. 1: MTA at bl.**	**1.17 ± 0.61**	**1.42 ± 0.53**	**1.55 ± 0.63**	**0.0799**
SCD only	1.08 ± 0.58	1.38 ± 0.48	1.23 ± 0.39	0.4145
MCI only	1.46 ± 0.63	1.45 ± 0.57	1.90 ± 0.66	0.2497
**Rad. 1: ΔMTA/year**	**0.05 ± 0.08**	**0.06 ± 0.06**	**0.11 ± 0.10**	**<.0001**
SCD only	0.05 ± 0.08	0.04 ± 0.05	0.08 ± 0.10	0.8349
MCI only	0.05 ± 0.07	0.07 ± 0.06	0.14 ± 0.08	<.0001
**Rad. 2: MTA at bl.**	**0.53 ± 0.70**	**0.56 ± 0.68**	**0.71 ± 0.78**	**0.6273**
SCD only	0.44 ± 0.67	0.56 ± 0.68	0.36 ± 0.53	0.8233
MCI only	0.83 ± 0.69	0.55 ± 0.69	1.10 ± 0.83	0.2714
**Rad. 2: ΔMTA/year**	**0.06 ± 0.08**	**0.12 ± 0.07**	**0.14 ± 0.11**	**<.0001**
SCD only	0.06 ± 0.08	0.07 ± 0.05	0.11 ± 0.10	0.0111
MCI only	0.05 ± 0.07	0.15 ± 0.07	0.18 ± 0.11	<.0001
**AVRA: MTA at bl.**	**1.26 ± 0.54**	**1.40 ± 0.65**	**1.24 ± 0.58**	**0.5856**
SCD only	1.21 ± 0.51	1.22 ± 0.59	1.01 ± 0.42	0.4747
MCI only	1.44 ± 0.58	1.54 ± 0.66	1.48 ± 0.63	0.9687
**AVRA: ΔMTA/year**	**0.04 ± 0.04**	**0.07 ± 0.05**	**0.12 ± 0.08**	**<.0001**
SCD only	0.04 ± 0.04	0.07 ± 0.05	0.10 ± 0.08	<.0001
MCI only	0.05 ± 0.03	0.08 ± 0.04	0.15 ± 0.06	<.0001
**HC vol at bl. (mm^3)**	**3691 ± 454**	**3766 ± 553**	**3382 ± 480**	**0.0795**
SCD only	3735 ± 434	4011 ± 558	3595 ± 307	0.2906
MCI only	3547 ± 486	3549 ± 448	3190 ± 525	0.3072
**ΔHC/year (mm^3/year)**	**-37.8 ± 24.1**	**-54.4 ± 25.8**	**-96.4 ± 36.1**	**<.0001**
SCD only	-35.2 ± 24.7	-41.4 ± 20.0	-83.6 ± 22.3	<.0001
MCI only	-46.1 ± 19.9	-66.0 ± 24.8	-107.8 ± 41.8	<.0001
**ΔHC/year (%/year)**	**-1.1 ± 0.8**	**-1.5 ± 0.8**	**-2.9 ± 1.1**	—
**ILV vol at bl. (mm^3)**	**751 ± 504**	**956 ± 656**	**837 ± 438**	**0.3311**
SCD only	691 ± 453	821 ± 655	675 ± 268	0.8606
MCI only	943 ± 603	1077 ± 633	983 ± 505	0.7803
**ΔILV/year (mm^3/year)**	**38.5 ± 39.9**	**83.6 ± 82.4**	**112.4 ± 76.5**	**<.0001**
SCD only	37.0 ± 42.7	84.3 ± 93.2	68.0 ± 55.4	<.0001
MCI only	43.4 ± 28.6	83.0 ± 71.5	152.4 ± 70.7	<.0001
**ΔILV/year (%/year)**	**4.5 ± 3.5**	**8.8 ± 5.3**	**14.4 ± 9.4**	—
**ΔMMSE/year**	**-0.15 ± 0.47**	**-0.49 ± 0.70**	**-1.13 ± 1.02**	**<.0001**
SCD only	-0.05 ± 0.30	-0.19 ± 0.34	-0.87 ± 1.05	<.0001
MCI only	-0.53 ± 0.71	-0.74 ± 0.82	-1.41 ± 0.92	<.0001
**ΔADAS-DWR/year**	**-0.04 ± 0.38**	**0.14 ± 0.40**	**0.39 ± 0.50**	**<.0001**
SCD only	-0.03 ± 0.31	0.01 ± 0.40	0.49 ± 0.62	<.0001
MCI only	-0.07 ± 0.57	0.25 ± 0.36	0.28 ± 0.26	0.0003
